# Impact of FilmArray Gastrointestinal Panel Compared to Standard-of-Care Diagnostic Tests in Clinical Practice of Acute Gastroenteritis in an HIV Reference Center with Limited Resources †

**DOI:** 10.3390/diagnostics16010121

**Published:** 2026-01-01

**Authors:** Guilherme Alves de Lima Henn, Marina Farrel Côrtes, Pedro Pinheiro de Negreiros Bessa, Francisco Breno Ponte de Matos, Jacqueline Sousa, Juliana Festa Ortega

**Affiliations:** 1 Hospital São José de Doenças Infecciosas, Fortaleza 60455-610, Brazil; pinheirobessa@gmail.com (P.P.d.N.B.); fcobreno18@gmail.com (F.B.P.d.M.); 2 Instituto de Medicina Tropical, Departamento de Doenças Infecciosas e Parasitarias, Faculdade de Medicina, Universidade de São Paulo, São Paulo 01246-903, Brazil; marinafarrel@yahoo.com.br; 3 BioMérieux, São Paulo 04028-001, Brazil; jacqueline.sousa@biomerieux.com (J.S.); juliana.ortega@biomerieux.com (J.F.O.)

**Keywords:** FilmArray gastrointestinal, gastroenteritis, molecular diagnostic, standard of care, antimicrobial stewardship

## Abstract

**Background/Objectives:** Gastroenteritis remains a major global health concern, particularly in resource-limited regions, where rapid and accurate diagnosis is crucial for effective patient management. Syndromic multiplex PCR panels, such as the FilmArray gastrointestinal (FAGI) panel, offer the potential to significantly improve diagnostic yield and turnaround time, enabling more targeted treatments and reducing unnecessary antibiotic use. However, real-world data on their performance in low-resource settings remains scarce. This study evaluates the performance, clinical impact, and cost-effectiveness of the FAGI panel compared to standard of care (SOC) diagnostic methods in gastroenteritis cases at São José Hospital for Infectious Diseases in Fortaleza, Brazil, an HIV Reference Center, in a resource-limited region of a middle-income country. **Methods:** A retrospective observational study was conducted among patients tested with FAGI (n = 161) and a retrospective control group tested only with SOC methods (n = 166). **Results:** The FAGI panel was associated with a significant reduction in the turnaround time, antimicrobial use, and total treatment costs while increasing the pathogen detection rate. Specifically, the median diagnostic time was reduced by 18%, with an increase in pathogen detection compared to SOC methods (64% positivity compared to 32%). Moreover, the FAGI group experienced a 30% reduction in antibiotic use, with a corresponding 83% reduction in antimicrobial costs. **Conclusions:** These results suggest that the FilmArray panel may offer substantial benefits in terms of efficiency and cost savings, highlighting its potential for broader implementation in clinical practice, especially in resource-limited settings, to improve patient outcomes in infectious disease management.

## 1. Introduction

Gastroenteritis remains a significant global health concern as one of the leading causes of mortality, particularly in children [1]. Annually, around 1.31 million deaths are related to diarrhea, with a substantial burden on children under five years old, who account for nearly 499,000 of these deaths [2]. Its cause encompasses a spectrum of agents/products, including viruses, bacteria, parasites, and toxins. The most frequent etiological agents include noroviruses, which alone cause an estimated 210,000 deaths and 685 million diseases each year [3]. Additionally, Rotavirus, *Shigella* spp. and *Salmonella* spp. are also highly prevalent [2]. Viruses are usually the cause of around 25% of gastroenteritis cases [4,5]. Children under five years are disproportionately affected, with viral gastroenteritis accounting for more than 70% of cases in this population [5].

The prognosis of gastroenteritis is notably worse in low and middle-income countries due to factors like malnutrition and inadequate sanitation [2]. Despite the high morbidity and mortality, improvements in sanitation and vaccination can reduce gastroenteritis-related deaths. Continued public health initiatives can further mitigate this burden. This scenario becomes even more critical among people living with HIV in these settings, where gastroenteritis is associated with disproportionately high morbidity and mortality and a broad diversity of atypical and opportunistic etiologies that demand specific diagnostic and therapeutic approaches. In low-resource contexts, up to 90% of HIV-infected patients may experience diarrheal episodes during the course of disease, with intestinal parasites playing a central role [6].

The diagnosis routinely relies on clinical evaluation, laboratory tests, and imaging techniques [6]. Rapid diagnosis is linked to a better patient prognosis, as early microbial diagnosis allows for prompt initiation of targeted therapies that can significantly reduce morbidity and mortality [7], as well as avoid or limit inappropriate antibiotic prescription reducing unnecessary use of broad-spectrum antibiotics, ultimately combating antibiotic resistance effectively. This is essential for managing infections that can lead to severe complications, especially in vulnerable populations [8]. Delays in identifying the specific pathogen and its resistance profile are an important factor in treatment failure. This uncertainty forces clinicians to prescribe broad-spectrum antibiotics as a precaution. When the correct antibiotic is not administered in time, patient outcomes worsen, leading to increased mortality, longer hospital stays, higher healthcare costs, and increase bacterial resistance [9,10]. Additionally, rapid diagnostic tests are crucial for early case identification and surveillance during outbreaks, as emphasized in the 100 Days Mission initiative, a global initiative that aims to prepare the world for the next epidemic/pandemic [11].

The FilmArray Gastrointestinal (FAGI) panel is a syndromic multiplex PCR that detects common infectious diarrhea pathogens, including bacteria, parasites, and viruses. Given the importance of rapid and accurate diagnostics to improve clinical outcomes, the introduction of advanced diagnostic tools such as the FilmArray panel could have a significant impact on clinical practice, especially in hospital settings where the burden of infectious diseases is high [12]. Comparing their effectiveness and cost-effectiveness with conventional Standard of care (SOC) methods is essential to validate their large-scale implementation.

Although several studies have evaluated the effectiveness of FilmArray compared to traditional SOC diagnostic tests [13,14,15,16,17,18], this study aimed to evaluate its performance in a real-world setting, conducted in an HIV Reference Center from a major city with limited resources of a middle-income country. Furthermore, most of these studies were with a limited sample size. By focusing on the clinical and operational impact of the FilmArray panel, this study may contribute to improving diagnostic practices and patient outcomes in similar resource-limited environments.

## 2. Materials and Methods

*Study design*—This retrospective observational study was conducted at São José Hospital for Infectious Diseases, a tertiary care center in Fortaleza, Ceará, Brazil. We compared diagnostic yield, turnaround time (TAT), Days of Treatment (DOT), Length of stay (LOS) and total cost of treatment between patients who were tested with FilmArray GI panel (FAGI) (bioMérieux, Salt Lake City, UT, USA) (from June 2019 to November 2020) and a retrospective control group (from December 2017 to October 2018) of the same number of patients consecutively included, immediately prior to the panel being made available at the hospital. The same medical team was involved in the treatment of patients from both groups. Patients with a suspected gastrointestinal infection were enrolled after completing electronic medical records, regardless of whether the diarrheal syndrome was the cause of hospitalization or was contracted during hospitalization. Exclusion criteria were not having been hospitalized or lacking clinical or laboratory data in medical records. This study was approved by the local ethical committee, under the number CAAE 47310521.9.0000.5044. For the control group the above-mentioned inclusion and exclusion criteria and classification were maintained. In total, 332 patients were recruited, and 161 patients were included in the FAGI group and 166 in the control group.

*Laboratory tests*—BioFire FilmArray Gastrointestinal panel (FAGI—BioMerieux, Marcy L’Etoile, France) detects 22 pathogens in fecal swabs in about an hour. The pathogens include *Campylobacter* (*jejuni*, *coli* and *upsaliensis*), *Clostridioides difficile* (toxin A/B); *Plesiomonas shigelloides*, *Salmonella*, *Vibrio* (*parahaemolyticus*, *vulnificus* and *cholerae*), *Yersinia enterocolitica*; *Escherichia coli* O157, Enteroaggregative *Escherichia coli* (EAEC), Enteropathogenic *Escherichia coli* (EPEC), Enterotoxigenic *Escherichia coli* (ETEC) lt/st, Shiga-like toxin-producing *Escherichia coli* (STEC) stx1/stx2, *Shigella*/Enteroinvasive *Escherichia coli* (EIEC), *Cryptosporidium*; *C. cayetanensis*; *E. histolytica*; *G. lamblia*, Adenovirus F 40/41, Astrovirus; Norovirus GI/GII, Rotavirus A and Sapovirus (I, II, IV e V).

FAGI tests were performed according to the manufacturer’s instructions—out of the hospital of this study—at LACEN (Laboratório Central de Saúde Pública) which has limited opening hours (from Monday to Friday 8 a.m. to 4 p.m. and on weekends 10 a.m. to 12 p.m.). Standard of care (SOC) tests were performed according to the hospital’s routine tests available for gastroenteritis investigation. Those included parasitological examination of feces, stool culture, special stains for *Cystoisospora* and *Cryptosporidium* research, and *C. difficile* toxins A/B by enzyme-linked immunosorbent assay. No specific guidelines and restrictions were in place for clinicians regarding microbiological testing and therefore practice was at the clinician’s discretion. Therefore, some patients in both groups did not undergo these tests.

*Statistical analysis*—The SOC test was analyzed separately for the control group and the FAGI group, as well as collectively, considering the total number of SOC tests performed in both groups. The time from collection to results for the SOC tests was considered as the longest time for the test result. Treatment costs were calculated by multiplying the total number of antimicrobial doses used per patient by the cost per dose. Statistical analyses were performed using RStudio version 4.1.1. The Shapiro test was used to assess normality. Comparisons between groups were performed using a two-tailed Wilcoxon test for non-parametric continuous variables and a two-tailed Fisher’s exact test for binary categorical variables. A *p*-value of <0.05 was considered statistically significant. Graphics were generated with libraries ggsci, tidyverse, ggpubr, dplyr and irr and ggplot2.

## 3. Results

**Figure 1:**
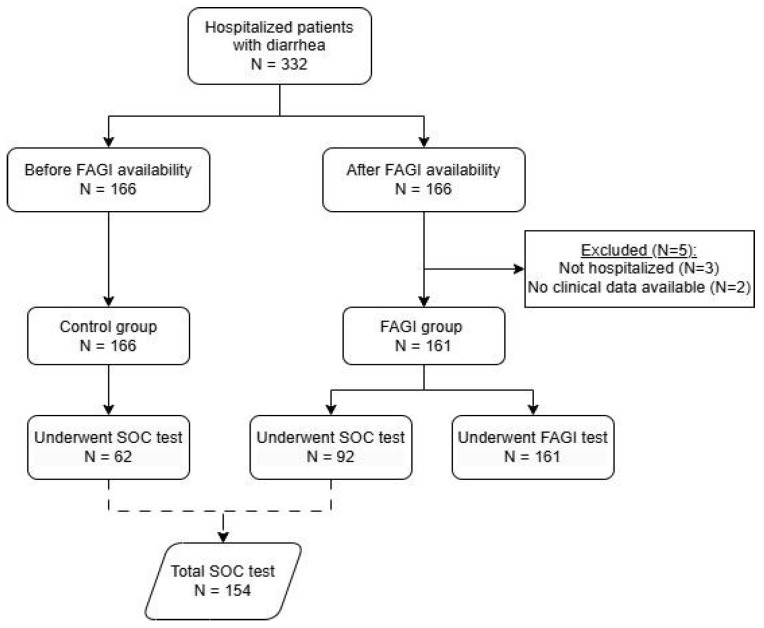
Flowchart of the study inclusion of patients. FAGI: FilmArray gastrointestinal panel; SOC: Standard of care. The dashed line indicates that the total number of SOC tests (N = 154) was obtained by combining patients from both groups who underwent SOC testing.

A total of 332 patients were divided into two groups: FAGI (n = 166, 153 adults and 13 children) and control (n = 166, 154 adults and 12 children). Five patients were excluded from the FAGI group: three were not hospitalized, and two lacked available clinical or laboratory data (Figure 1). The median age was 37 years (ranging from 6 months to 71 years) in the control group and 39 years (1 month to 87 years) in the FAGI group, with 38% and 28% of women, respectively. Most of the patients were positive for HIV, being 83% of the control group, and 75% of the FAGI group (*p* = 0.06). There were no significant differences between the groups in terms of mortality rate (*p* = 0.77) or LOS (*p* = 0.44). Notably, there was a significant 30% reduction in the antibiotic use in the FAGI group compared to the Control group (*p* < 0.001). The total number of antimicrobials used per patient was reduced by 50% (*p* < 0.001), with a 37% reduction in the overall antimicrobial use (*p* < 0.001). Patients in the FAGI group had a median of 5 days shorter duration of antimicrobial DOT (median days: 6 vs. 11, *p* < 0.001), 83% reduction in total antimicrobial costs (median total cost: BRL 4697 vs. BRL 27,174, *p* < 0.001), and an increase of 77% in the number of pathogens detected (median: 103 vs. 24, *p* < 0.001) compared to the control group (Supplementary Figure S1). It is important to note that one of the primary contributors to the substantial cost difference was the use of liposomal amphotericin/lipid complex for empirical therapy in some patients with suspected Histoplasma-related diarrhea. These findings are summarized in Table 1.

In the control group, 62 patients underwent SOC tests. In the FAGI group, all patients had FilmArray tests, and 92 also had SOC tests. Then we compared the results of the total number of patients that underwent SOC tests (n = 154) with FilmArray (n = 161). The use of FilmArray significantly increased the number of samples with a pathogen identified, with 64% positivity compared to 32% in those tested with SOC methods (*p* < 0.001). The median number of pathogens detected per sample was also higher in the FilmArray group, with a median of 2 pathogens (range 1–5), compared to a median of 0 (range 0–2) for the SOC test (*p* < 0.001). Furthermore, FilmArray reduced the TAT from sample collection to diagnostic result by 18%, with a median time of 60 h (range 5–500), compared to 73 h (range 12–394; *p* < 0.001) for SOC tests. When the time from sample shipping to result was evaluated, FilmArray reduced the time by 38% with a median time of 45 h (range 5–500; *p* < 0.001). These results are summarized in Table 2 and Supplementary Figure S2.

**Figure 2:**
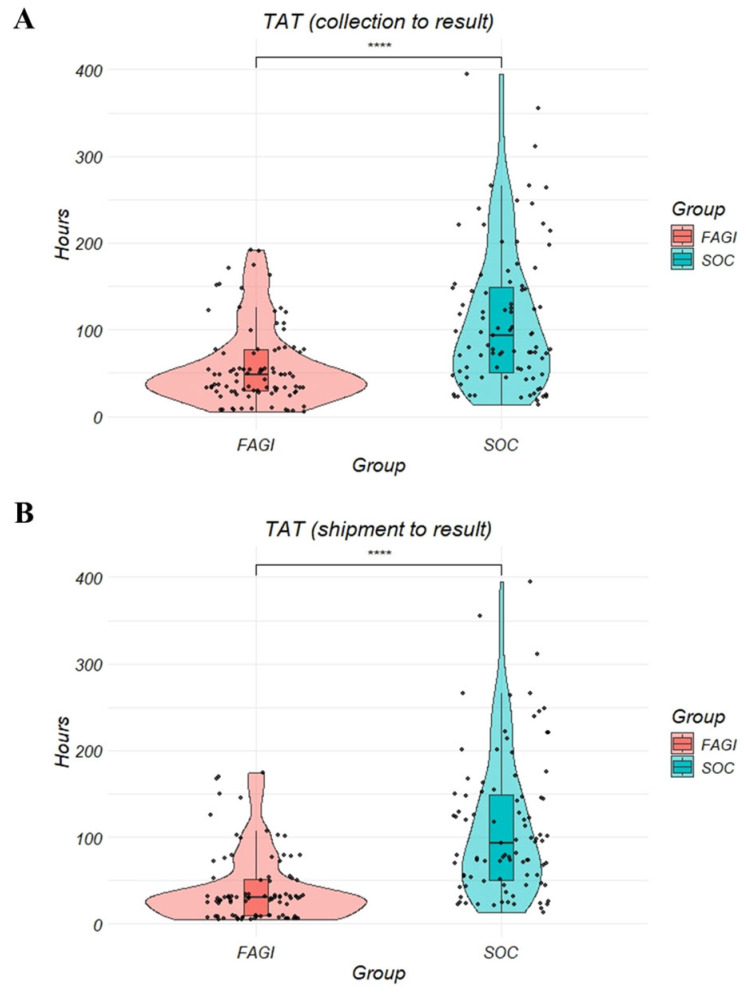
Turnaround time (TAT) of FilmArray compared to Standard of care (SOC) tests from patients that underwent both methods (n = 92). (**A**) Time from sample collection to results. (**B**) Time from sample shipment to result. **** = *p* < 0.0001.

Next, to compare the turnaround times (TAT) of both diagnostic methods, we restricted our analysis to the subgroup of patients who underwent both FilmArray and SOC testing. This approach ensured a direct comparison by eliminating potential confounding factors from patients who received only one of the tests. Among the 92 patients from the FAGI group, FAGI demonstrated a 45h shorter TAT (median hours: 48 vs. 93, *p* < 0.001) considering the collection time to result and a 63h shorter TAT (median hours: 30 vs. 93, *p* < 0.001) considering the shipment time to result (Figure 2) compared to SOC tests.

**Figure 3:**
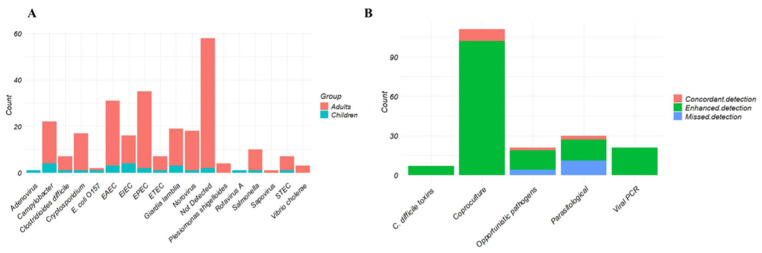
(**A**) Overall FilmArray pathogen detection of the 161 patients from FAGI group. (**B**) Agreement of FilmArray detection compared to SOC methods of the 92 patients from the FAGI group with a SOC diagnostic test.

FilmArray identified 217 targets in the FAGI group. The most common were EPEC (n = 35), EAEC (n = 31), *Campylobacter* (n = 22), *Giardia lamblia* (n = 19) and Norovirus (n = 18). There were 58 samples without a pathogen detected by FilmArray, with a positivity rate of 64% (62% in adults and 85% in pediatric patients; Figure 3A, Table 3). Additionally, we observed an 88% higher pathogen detection rate for FilmArray. There were 201 pathogens detected only by FAGI, 15 detected only by SOC and 14 identified by both methods Table 3. However, there was one missed detection of Entamoeba histolytica in one patient. Moreover, some parasites (*Ascaris lumbricoides*, *Endolimax nana*, *Entamoeba coli*, Hookworm and *Cystoisospora belli*) not included in the FAGI panel were also missed (Figure 3B, Table 3).

## 4. Discussion

The findings of this study indicate that the FilmArray Gastrointestinal (FAGI) panel significantly enhances the ability to diagnose gastroenteritis compared to SOC methods. The study provides valuable insights into the challenges and opportunities associated with implementing advanced diagnostic technologies in settings where healthcare resources are scarce. Patients in the FAGI group had a shorter duration of antimicrobial treatment, reduced treatment costs, and a higher number of pathogens detected compared to those who underwent SOC testing, highlighting the advantages of molecular diagnostic methods.

Several studies support the efficacy of multiplex PCR panels like FilmArray in improving diagnostic accuracy and clinical outcomes. Beal et al. demonstrated that the use of the FilmArray panel reduced the time from stool collection to discharge by 15%, which consequently shortened the total health care costs by $293.61 per patient [19]. Similarly, Cybulski et al. found that FilmArray improved the sample detection of pathogens 6 times (35.3% of specimens, compared to 6.0% for culture) in oncology patients. Also, the median time from collection to result was 18 h for FilmArray and 47 h for culture [12]. Additionally, they found a reduction of 50 h from collection to initiation of antimicrobial therapy leading to more targeted and effective treatments. Indeed, other studies also concluded that FilmArray can positively impact the medical management of gastroenteritis [17,20,21]. Another study showed that FilmArray provides results in a median time of 2.2 h compared to 77.5 h for culture methods [22]. Despite the significant reduction observed with the use of the FilmArray Gastrointestinal panel, we did not find such a drastic reduction in diagnostic time (60 h vs. 73 h) as expected. It is important to note that in this study, the FilmArray was not available in the hospital’s routine laboratory. The samples had to be sent to an external laboratory, which subjected them to transportation time and the laboratory’s availability to perform the tests. This logistical process, which included weekends and holidays when the reference laboratory was not operating, contributed significantly to the increase in total diagnostic time. Although the test itself takes only one hour to complete, external factors considerably delayed the delivery of results. Even so, we evidenced a major impact on the costs of treatment and use of antimicrobials.

The median hospitalization duration for the FAGI group was 13 days (ranging from 1 to 114 days), compared to 12 days (ranging from 1 to 110 days) for the control group. However, it has been previously evidenced by Torres-Miranda et al. that hospital LOS was shorter (3 vs. 7.5 days) for the FilmArray group compared to standard methods [20]. Accordingly, Carmon et al., evidenced a reduction in LOS in patients with a positive FilmArray gastrointestinal result (2 days) compared to negative test results [17]. DelRusso et al. found a reduction of 1.6 days in LOS. Although this difference was not statistically significant (*p* = 0.13), they also observed a reduction of 24 h in time to de-escalation (*p* < 0.001) and a reduction of 30h in TAT (*p* < 0.001) [23]. It is important to highlight that this study period coincided with the COVID-19 pandemic, during which many cases experienced prolonged hospital stays due to complications related to COVID-19 [24]. This context made it challenging to directly compare the FAGI group with the historical control group, which was observed in the year before the pandemic.

We observed a reduction in antimicrobial use per patient of 37% in the FAGI group compared to the control. This reduction aligns with the goals of antimicrobial stewardship programs, which aim to optimize antibiotic use and reduce resistance [25,26]. The use of molecular diagnostic tests such as FAGI in clinical practice can decrease the unnecessary use of antibiotics [14,27]. By providing rapid and accurate results, FilmArray enables clinicians to make informed decisions about antibiotic prescriptions, thereby minimizing unnecessary broad-spectrum antibiotic use. Moreover, the benefits of using FilmArray are notable. The reduction in the length of hospital stays, seen by other authors, and the shorter duration of antimicrobial treatment may contribute to decreased overall healthcare costs. Although we have not evaluated overall costs, the reduction and shorter duration of antimicrobial treatment may contribute to decreased total healthcare costs.

The economic impact is also a crucial factor. Although the initial costs of multiplex molecular testing may be higher, the reduction in overall treatment costs and the shortened length of hospital stay may justify the costs in the long term [28]. Ambrosius-Eichner et al. (2024) [22] showed that FilmArray GI panel significantly improves pathogen detection rates (39.9% vs. 15% with SOC methods). Our study reinforces these observations, showing that FAGI is not only more time-efficient but may also be cost-effective by reducing the costs of antimicrobial treatment by 83%.

Studies like this are crucial in assessing the impact of molecular diagnostics, such as multiplex PCR panels, in low- and middle-income countries like Brazil. These advanced diagnostic tools, while often expensive, have the potential to significantly improve clinical outcomes through rapid and accurate pathogen detection. However, a limitation of our study is that we could not comprehensively evaluate the total hospitalization costs, including hospital and diagnostic testing expenses. This gap underscores the need for further research to substantiate the cost-effectiveness of high-priced diagnostic kits like the FilmArray in resource-limited settings. While the upfront costs of multiplex PCR panels may be higher, the long-term savings and improved patient outcomes could justify their broader adoption.

The FilmArray GI Panel provides faster results, which is crucial for timely treatment decisions in patients with gastroenteritis [21,27,29]. Despite its advantages, there are concerns regarding the potential for false positives and the appropriateness of testing in late-stage hospital admissions, suggesting a need for diagnostic stewardship [18]. Other limitations of this study include its retrospective design, which does not allow for proper randomization of patients based on their comorbidities or standardization of the attending physicians’ practices regarding the collection of SOC tests and the prescription of antimicrobials. The main limitation for generalizing the results of this study is that the sample consisted predominantly of people living with HIV, which represents a very specific clinical profile. In this population, diarrhea may be related not only to the etiological agent of acute gastroenteritis under investigation but also to multiple concurrent factors, such as HIV itself (HIV-associated enteropathy), rarely adverse effects of currently used antiretroviral drugs, and the concomitant presence of gastrointestinal opportunistic infections. This set of potential causes of diarrhea, often simultaneous or overlapping, reduces the external validity of the study, making it difficult to extrapolate the findings to populations without HIV or with lower degrees of immunosuppression, in which the etiological profile, clinical course, and therapeutic response may differ substantially. However, it should be noted that the medical team remained consistent throughout the study period, which may mitigate some variability in clinical practices but does not eliminate potential biases.

It is important to note that the fact that this study was conducted in an infectious disease hospital likely contributed to limiting the inappropriate use of antimicrobials, given the specialized focus of the institution. To achieve similar results in general hospitals, it is essential that the interpretation of the panel results and subsequent clinical decisions be evaluated and defined in collaboration with an infectious diseases team.

In conclusion, this study underscores the clinical and economic advantages of the FilmArray Gastrointestinal panel over conventional diagnostic methods for gastroenteritis. The implementation of such molecular diagnostics can enhance patient care by enabling timely, accurate diagnosis and more effective treatment strategies.

## Figures and Tables

**Table 1:** Cohort description with sociodemographic and clinical data of the control and FAGI groups.

	Control Group		FAGI		*p* Value
	n or Median (% or Variation)		n or Median (% or Variation)		
Total	166	(100)	161	(100)	-
Age Median (years)	37	(0.5–71)	39	(0.1–87)	0.04
Female	63	(38)	45	(28)	0.07
HIV infected	138	(83)	120	(75)	0.06
Outcomes *					
Mortality	19	(11)	20	(12)	0.77
Hospital length of stay (LOS)	12	(1–110)	13	(1–114)	0.44
Patients with antibiotic use	133	(80)	81	(50)	<0.001
Patients with any antimicrobial use	166	(100)	102	(63)	<0.001
Number of antimicrobials per patient	2	(1–12)	1	(0–4)	<0.001
Total days (sum) of antimicrobial use	2323	(2–121)	1232	(0–45)	-
Antimicrobial days per patient	11	(2–121)	6	(0–45)	<0.001
Total (sum) antimicrobial cost (BRL)	27,174	(1.4–7848)	4697	(0–576)	-
Antimicrobial cost per patient (BRL)	21	(1.4–7848)	2	(0–576)	<0.001

* Antibiotic drug refers to those intended to treat bacteria while antimicrobial drugs may be intended to treat any type of microorganism, including parasites, viruses, and fungi.

**Table 2:** Laboratory variables of patients from FAGI and control groups. Patients from both groups who had Standard of care (SOC) tests collected were added to the “SOC Test” column. Each test is also described separately in the “Coproculture”, “Parasitological” and “Screening for Opportunistic Pathogens” columns.

	FAGI-FilmArray		Total SOC Test		*p* Value	Coproculture		Parasitological		Opportunist Screening	
	n or Median (% or Variation)		n or Median (% or Variation)			n or Median (% or Variation)		n or Median (% or Variation)		n or Median (% or Variation)	
Total	161	(100)	154	(100)	-	77	(50)	109	(70.8)	42	(27.3)
Sample with pathogen detected	103	(64)	50	(32)	<0.001	23	(30)	41	(38)	21	(50)
N of pathogens detected	2	(1–5)	0	(0–2)	<0.001	0	(0–2)	0	(0–2)	1	(0–1)
Time from collection to result (hours)	60	(5–500)	73	(12–394)	<0.001	98	(25–394)	27	(12–311)	70	(13–246)
Time from shipping to result (hours)	45	(5–500)	-	(-)	<0.001	-	(-)	-	(-)	-	(-)

**Table 3:** Pathogen detection of FAGI (FilmArray gastrointestinal) compared with SOC (Standard of care) methods.

		Total (n = 216)	FilmArray Only	SOC Only	Co-Identification
Adenovirus		1	1	-	-
*Ascaris lumbricoides*		2	-	2	-
*C. difficile*		7	7	0	0
*Campylobacter*		22	22	0	0
*Cryptosporidium*		17	15	0	2
*Escherichia coli* (total):		98	95	0	3
	*E. coli* O157	2	2	0	
	EAEC	31	31	0	
	EPEC	35	34	0	1
	ETEC	7	7	0	
	STEC	7	7	0	
	EIEC/*Shigella*	16	14	0	2
*Entamoeba coli/hystolitica*		2	0	2	0
*Endolimax nana*		5	-	5	-
*Giardia Lambia*		19	16	0	3
Hookworm		2	-	2	-
*Cytoisospora belli*		4	-	4	-
Norovirus		18	18	-	-
*Plesiomonas shigelloides*		4	4	0	0
Rotavirus		1	1	-	-
*Salmonella*		10	5	0	5
Sapovirus		1	1	-	-
*Vibrio cholerae*		3	2	0	1

## Data Availability

The original contributions presented in this study are included in the article/Supplementary Material. Further inquiries can be directed to the corresponding author.

## Supplementary Materials

The following supporting information can be downloaded at: https://www.mdpi.com/article/10.3390/diagnostics16010121/s1, Supplementary Figure S1: Hospitalization comparison between groups. Supplementary Figure S2: Laboratory variables comparison between patients that underwent FilmArray.

## Abbreviations

The following abbreviations are used in this manuscript:

FAGI	FilmArray gastrointestinal panel
SOC	Standard of care
TAT	Turnaround time
DOT	Days of Treatment
LOS	Length of stay
LACEN	Laboratório Central de Saúde Pública
