# Supplementary material for: Impact of FilmArray Gastrointestinal Panel Compared to Standard-of-Care Diagnostic Tests in Clinical Practice of Acute Gastroenteritis in an HIV Reference Center with Limited Resources
Source: Diagnostics (Basel). 2026 Jan 1;16(1):121. doi: 10.3390/diagnostics16010121 (PMC12786060; doi:10.3390/diagnostics16010121)
Supplement: Supplementary file 1 [file diagnostics-16-00121-s001.zip › diagnostics-3961475-supplementary.pdf]

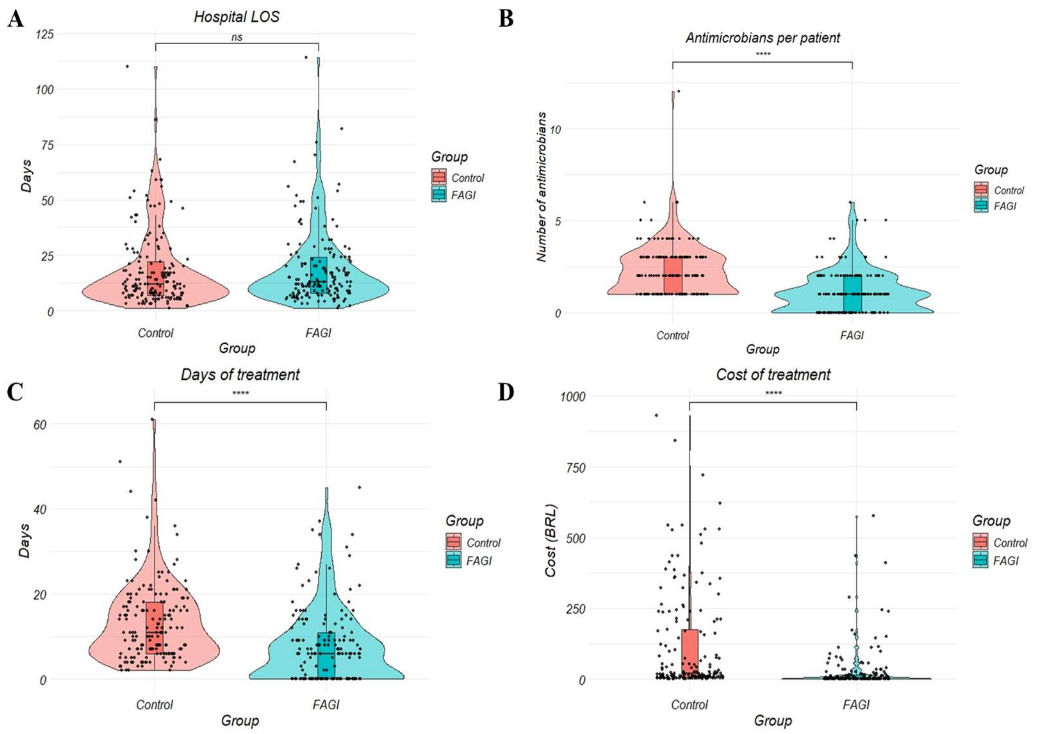

Supplementary Figure S1: Hospitalization comparison between FAGI (n = 161, blue) and control (n = 166, red) groups. **A**. Total Hospital Length of stay (LOS); **B**. Number of antimicrobials per patient; **C**. Total days of treatment; **D**. Total cost of treatment. ns = non-significant; **** = p<0.0001

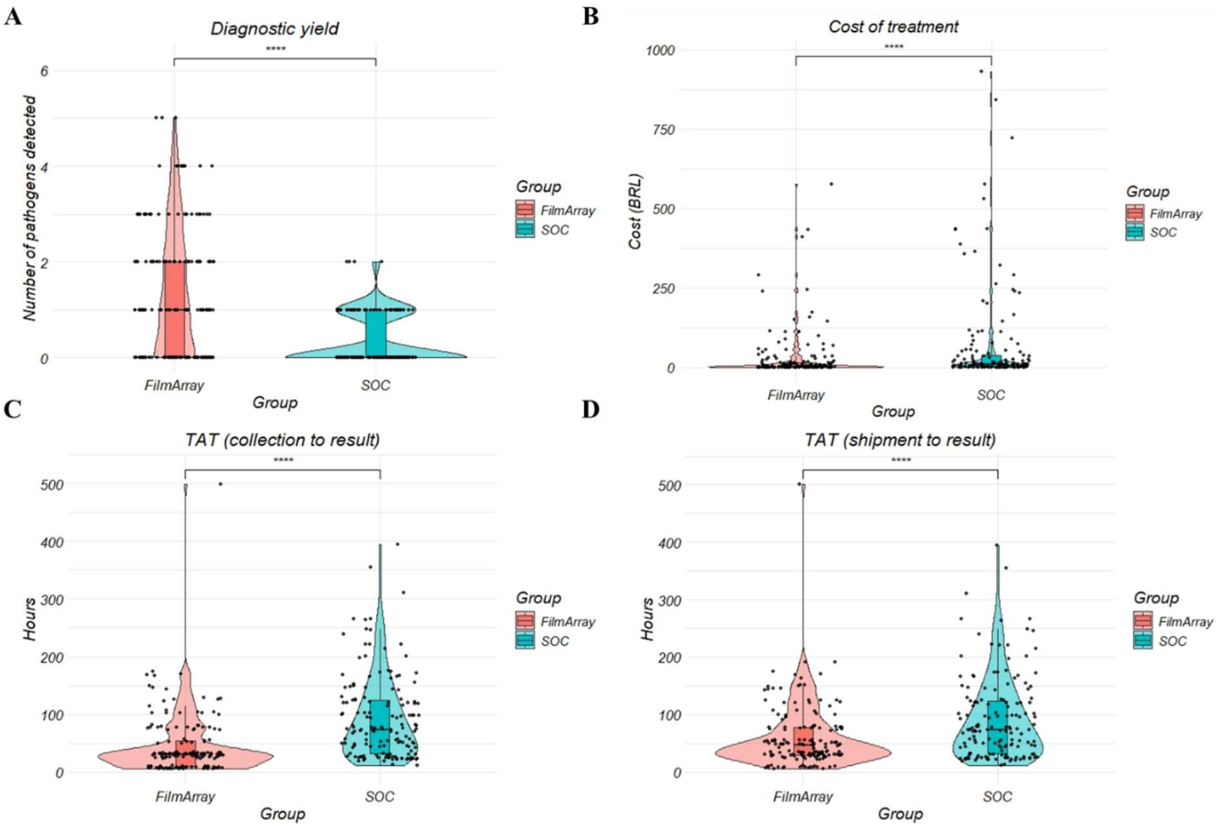

Supplementary Figure S2: Laboratory variables comparison between patients that underwent FilmArray (n = 161; blue) and Standard of care (SOC) tests (n= 154; red). **A**. Diagnostic yield

measured by the total of pathogens detected; **B**. Total treatment costs; **C**. Turnaround time (TAT) measured by the time from sample collection to result; **D**. Turnaround time (TAT) measured by the time from sample shipment to result. **** = $p<0.0001$
